# MARS: Mutation-Adjusted Risk Score for Advanced Systemic Mastocytosis

**DOI:** 10.1200/JCO.19.00640

**Published:** 2019-09-11

**Authors:** Mohamad Jawhar, Juliana Schwaab, Iván Álvarez-Twose, Khalid Shoumariyeh, Nicole Naumann, Johannes Lübke, Cecelia Perkins, Javier I. Muñoz-González, Manja Meggendorfer, Vanessa Kennedy, Georgia Metzgeroth, Alice Fabarius, Dietmar Pfeifer, Karl Sotlar, Hans-Peter Horny, Nikolas von Bubnoff, Torsten Haferlach, Nicholas C.P. Cross, Wolf-Karsten Hofmann, Wolfgang R. Sperr, Andrés C. García-Montero, Peter Valent, Jason Gotlib, Alberto Orfao, Andreas Reiter

**Affiliations:** ^1^ University Hospital Mannheim, Heidelberg University, Mannheim, Germany; ^2^ Spanish Network on Mastocytosis, Toledo and Salamanca, Spain; ^3^ Virgen del Valle Hospital, Toledo, Spain; ^4^ University of Freiburg, Freiburg, Germany; ^5^ Stanford University School of Medicine/Stanford Cancer Institute, Stanford, CA; ^6^ University of Salamanca and Biomedical Research Institute of Salamanca, Salamanca, Spain; ^7^ Munich Leukemia Laboratory, Munich, Germany; ^8^ Paracelsus Medical University of Salzburg, Salzburg, Austria; ^9^ Ludwig-Maximilians-University, Munich, Germany; ^10^ Wessex Regional Genetics Laboratory, Salisbury, United Kingdom; ^11^ University of Southampton, Southampton, United Kingdom; ^12^ Medical University of Vienna, Vienna, Austria

## Abstract

**PURPOSE:**

To develop a risk score for patients with advanced systemic mastocytosis (AdvSM) that integrates clinical and mutation characteristics.

**PATIENTS AND METHODS:**

The study included 383 patients with AdvSM from the German Registry on Disorders of Eosinophils and Mast Cells (training set; n = 231) and several centers for mastocytosis in the United States and Europe, all within the European Competence Network on Mastocytosis (validation set; n = 152). A Cox multivariable model was used to select variables that were predictive of overall survival (OS).

**RESULTS:**

In multivariable analysis, the following risk factors were identified as being associated with OS: age greater than 60 years, anemia (hemoglobin < 10 g/dL), thrombocytopenia (platelets < 100 × 10^9^/L), presence of one high molecular risk gene mutation (ie, in *SRSF2*, *ASXL1*, and/or *RUNX1*), and presence of two or more high molecular risk gene mutations. By assigning hazard ratio–weighted points to these variables, the following three risk categories were defined: low risk (median OS, not reached), intermediate risk (median OS, 3.9 years; 95% CI, 2.1 to 5.7 years), and high risk (median OS, 1.9 years; 95% CI, 1.3 to 2.6 years; *P* < .001). The mutation-adjusted risk score (MARS) was independent of the WHO classification and was confirmed in the independent validation set. During a median follow-up time of 2.2 years (range, 0 to 23 years), 63 (16%) of 383 patients experienced a leukemic transformation to secondary mast cell leukemia (32%) or secondary acute myeloid leukemia (68%). The MARS was also predictive for leukemia-free survival (*P* < .001).

**CONCLUSION:**

The MARS is a validated, five-parameter, WHO-independent prognostic score that defines three risk groups among patients with AdvSM and may improve up-front treatment stratification for these rare hematologic neoplasms.

## INTRODUCTION

Systemic mastocytosis (SM) is characterized by expansion of clonal mast cells that infiltrate various organ systems. The extent of organ infiltration and subsequent organ damage serve as a basis for the WHO classification of SM as indolent SM or advanced SM (AdvSM). AdvSM includes patients with SM and an associated hematologic neoplasm (SM-AHN), aggressive SM (ASM), and mast cell leukemia (MCL).^1-4^

SM-AHN (70% to 80% of all patients with AdvSM) is the most heterogeneous and clinically challenging subtype. The AHN usually resembles a myeloid neoplasm (eg, chronic myelomonocytic leukemia, myelodysplastic/myeloproliferative neoplasm unclassifiable, chronic eosinophilic leukemia, or myelodysplastic syndrome). In the vast majority of patients, the phenotypically most important somatic mutation (ie, *KIT* D816V) is detectable in the clonal mast cell compartment and in cells derived from the AHN.^5,6^

The WHO classification is most widely used for prognostication and has been validated in multiple studies. In contrast to indolent SM, AdvSM has a poor prognosis.^7^ The overall survival (OS) of patients with AdvSM ranges from a few months to several years, with a median OS of approximately 4 years.^8,9^

A number of clinical, serologic, cytomorphologic, immunologic, and molecular parameters have been reported to be of (WHO-independent) prognostic significance in patients with AdvSM.^10,11^ Recent data, however, have highlighted that the molecular landscape of AdvSM is complex, with at least one somatic mutation in addition to KIT D816V (eg, in *ASXL1*, *CBL*, *JAK2*, *RUNX1*, *SRSF2*, or *TET2*) being present in more than 60% of patients with AdvSM.^12,13^ In more recent studies, several groups examined the prognostic impact of these mutations. The presence and number of additional molecular mutations, notably in *SRSF2*, *ASXL*, and/or *RUNX1* (S/A/R), have a strong adverse influence on progression (leukemic transformation) to secondary MCL and/or secondary acute myeloid leukemia (AML), response to treatment, and OS.^8-10,13-15^ To date, the independent prognostic value of most variables and proposed risk scores has been derived from relatively small sets of patients, and they have not been confirmed or validated.^14^

In this study, we evaluated a large cohort of clinically, morphologically, and genetically well-characterized patients with AdvSM who were enrolled in the German Registry on Disorders of Eosinophils and Mast Cells with the aim to establish a risk score integrating both clinical and molecular characteristics. The proposed clinical risk score (CRS) and mutation-adjusted risk score (MARS) were subsequently validated in an independent cohort of patients with AdvSM derived from several centers within the European Competence Network on Mastocytosis (ECNM).

## PATIENTS AND METHODS

### Patients

A total of 383 patients with AdvSM were included. For the training set, 231 patients with AdvSM were recruited from the German Registry on Disorders of Eosinophils and Mast Cells between 2003 and 2018, with a final update performed in November 2018. The diagnosis of AdvSM (SM-AHN, ASM, or MCL) was established according to the WHO classification.^1,4^ For the training set, bone marrow (BM) biopsies and BM smears were evaluated by reference pathologists from the ECNM (H.-P.H. and K. Sotlar). The study design adhered to the tenets of the Declaration of Helsinki and was approved by the institutional review board of the Medical Faculty of Mannheim, Heidelberg University (Heidelberg, Germany). All patients gave written informed consent. The validation set included 152 patients from multiple centers of excellence for mastocytosis in the United States (Stanford, CA) and Europe (Spanish Network on Mastocytosis, Toledo and Salamanca, Spain; Vienna, Austria; and Freiburg, Germany – all members of the ECNM).

### Mutational and Cytogenetic Analyses

Molecular analyses were performed at diagnosis of AdvSM (prospectively or retrospectively). Targeted next-generation sequencing was performed by either 454 FLX amplicon chemistry (Roche, Penzberg, Germany) or library preparation on the basis of the TruSeq Custom Amplicon Low Input protocol (Illumina, San Diego, CA) and sequencing on the MiSeq instrument (Illumina) to investigate the mutation status of *KIT* and the following 32 genes: *ASXL1*, *BCOR*, *CALR*, *CBL*, *CSNK1A1*, *DNMT3A*, *ETNK1*, *ETV6*, *EZH2*, *FLT3*, *GATA1*, *GATA2*, *IDH1*, *IDH2*, *JAK2*, *KRAS*, *MLL*, *MPL*, *NPM1*, *NRAS*, *PHF6*, *PIGA*, *PTPN11*, *RUNX1*, *SETBP1*, *SF3B1*, *SRSF2*, *TET2*, *TP53*, *U2AF1, ZRSR2*, and *WT1*.^12^ Subsequent to bcl2fastq and demultiplexing, alignment and variant calling were performed using JSI SeqNext v4.4.0 (JSI Medical Systems, Kippenheim, Germany) software with default parameters. Only base calls with a quality score of greater than 30 were considered for additional processing. A median of approximately 1,800 reads were aligned to the target region. All regions below the minimal coverage of 400 reads were rejected and resequenced for higher depth. Variants were called with a variant allele frequency cutoff of 3%, and each was assessed manually for pathogenicity. Mutation assessment was performed using the Catalogue of Somatic Mutations in Cancer (v78), Single Nucleotide Polymorphism Database (v150), ClinVar (2018-07), Genome Aggregation Database (r2.0.2), and Database for Nonsynonymous Single Nucleotide Polymorphisms’ Functional Predictions (v3.5). Cytogenetic analysis and reporting were performed according to the International System for Human Cytogenetic Nomenclature criteria using standardized techniques.

### Statistical Analyses

All statistical analyses considered clinical and laboratory parameters obtained at the time of diagnosis or first referral to our center, which, in most instances, coincided with time of BM biopsy and study sample collection. OS analysis was considered from the date of diagnosis to date of death or last visit. Leukemia-free survival (LFS) was considered from the date of diagnosis to date of death, last visit, or progression (leukemic transformation) to secondary MCL or secondary AML. As the MARS reflected the highest concordance index (C-index), LFS analyses were examined for this score, only. OS probabilities and LFS were estimated using the Kaplan-Meier method and compared using the log-rank test in univariable analysis. For OS, a Cox proportional hazards model with a stepwise selection procedure was used to select covariates on the basis of their statistical significance (*P* < .05). Significant covariates were confirmed by forward-selection and backward-elimination techniques. On the basis of the magnitude of the hazard ratios (HRs) obtained from multivariable analysis, a weighted score was assigned to each significant variable for OS in the learning set. Bonferroni adjustments were made to univariable analysis with no changes to the multivariable models. The Mann-Whitney *U* test was used to compare continuous variables and medians of distributions. The receiver operating characteristic (ROC) curve was used to dichotomize continuous variables to define optimal cutoff values for each variable used in univariable analyses. Harrell’s C-index (on the basis of the ROC) was used to evaluate the ability of the risk scores to predict outcome (C-index measures the goodness of fit of a model, with 0.5 indicating no discrimination and 1.0 indicating perfect prediction). For categorical variables, two patient groups were compared using the Fisher’s exact test. All tests were two-sided, with *P* < .05 considered as statistically significant.

## RESULTS

### Characteristics

The characteristics of the training set patients (n = 231) are listed in Table 1. The median age was 69 years, and there was a male predominance (68%). The WHO diagnosis was ASM in 30 patients (13%), SM-AHN in 181 patients (78%), and MCL (with or without AHN) in 20 patients (9%). The four most common AHN subtypes were chronic myelomonocytic leukemia, myelodysplastic/myeloproliferative neoplasm unclassifiable, chronic eosinophilic leukemia, and myelodysplastic syndrome. The median leukocyte count, hemoglobin level, and platelet count were 8.3 × 10^9^/L, 10.3 g/dL, and 115 × 10^9^/L, respectively, and the median serum tryptase level was 168 µg/L (normal value < 11.4 µg/L). Treatment modalities included midostaurin, cladribine, and sequential midostaurin followed by cladribine or cladribine followed by midostaurin in 111 patients (48%). During a median follow-up time of 2.2 years (range, 0 to 23 years), 118 patients (51%) died. Transformation to secondary MCL (43%) or secondary AML (57%) was observed in 35 patients (15%; Table 1).

**TABLE 1.**
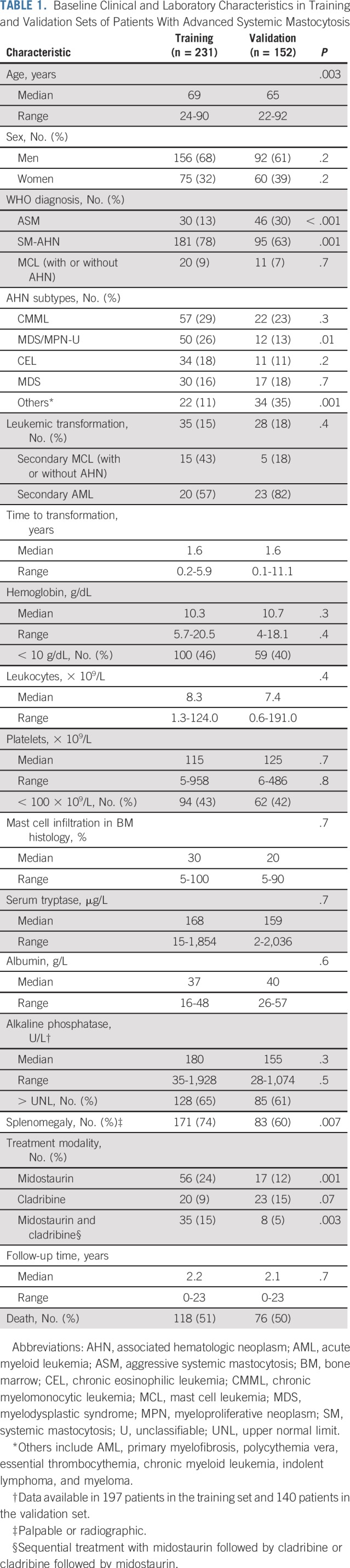
Baseline Clinical and Laboratory Characteristics in Training and Validation Sets of Patients With Advanced Systemic Mastocytosis

No significant differences were seen between the training set and the validation set (n = 152) regarding sex, hemoglobin level, platelet count, alkaline phosphatase level, leukemic transformation, median follow-up time, and number of deaths (Table 1). In the training set, compared with the validation set, patients were significantly older (median age, 69 *v* 65 years, respectively), ASM was less frequent (13% *v* 30%, respectively), and SM-AHN was more frequent (78% *v* 63%, respectively). Compared with the validation set, more patients in the training set were treated with midostaurin or sequential treatment with midostaurin followed by cladribine or cladribine followed by midostaurin.

**FIG A1.**
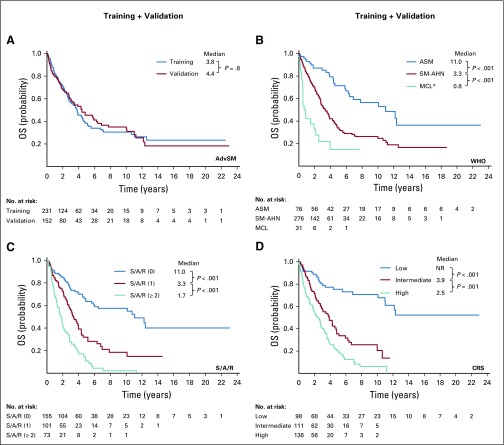
Overall survival (OS) of all patients with advanced systemic mastocytosis (AdvSM; training and validation sets) grouped by (A) AdvSM (includes all AdvSM subtypes, aggressive systemic mastocytosis [ASM], systemic mastocytosis with an associated hematologic neoplasm [SM-AHN], and mast cell leukemia [MCL]), (B) WHO-based stratification, (C) *SRSF2*, *ASXL1*, and *RUNX1* (S/A/R) mutation–based stratification, and (D) the clinical risk score (CRS). (*) The MCL cohort included patients with MCL with or without AHN. NR, not reached.

Importantly, the median OS and LFS times were not significantly different between the training and validation sets (OS, 3.8 and 4.4 years, respectively; *P* = .8; LFS, 3.3 and 3.5 years, respectively; *P* = .9; Appendix Fig A1A, online only). In addition, no differences were seen regarding OS among the four most common AHNs (Appendix Fig A3A-B, online only) and between *KIT*-positive and *KIT*-negative patients (Appendix Table A1, online only).

### Gene Mutations

**FIG 1.**
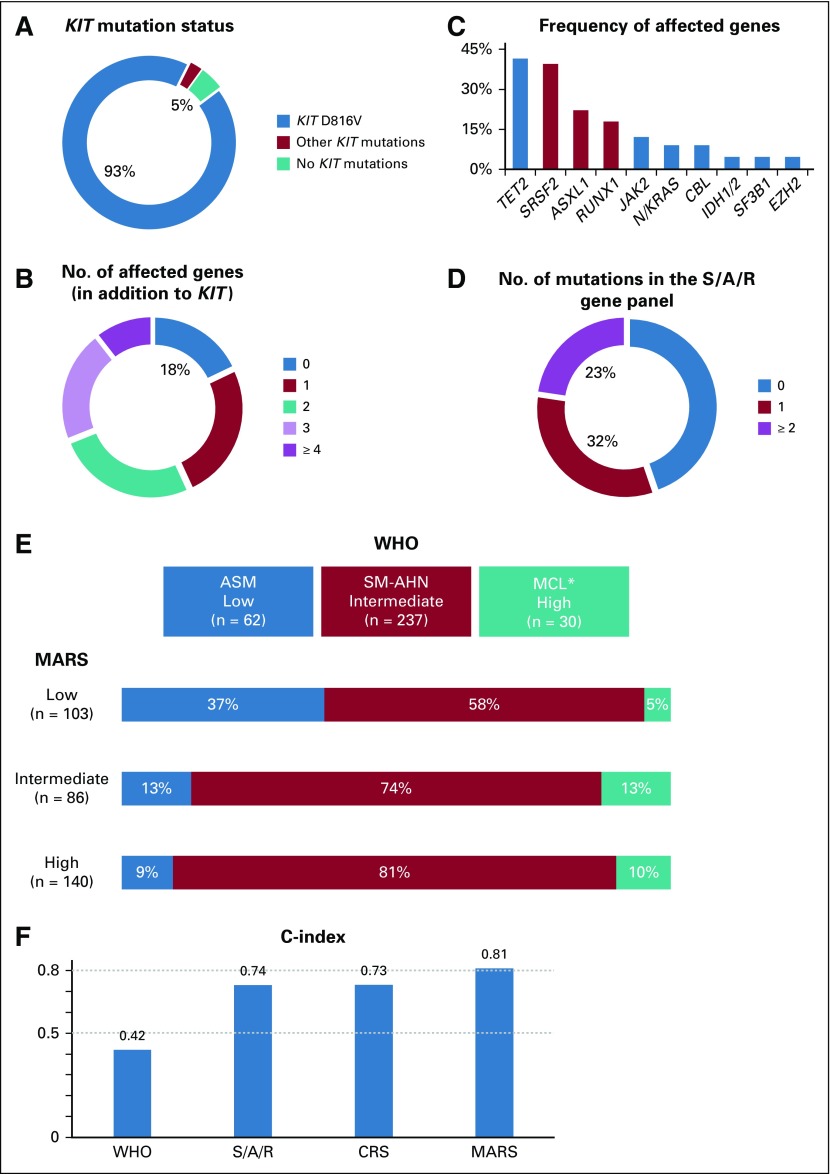
(A) Relative frequency distribution of *KIT* mutations, (B) number of affected genes in addition to *KIT*, (C) frequency of mutations in addition to *KIT*, and (D) number of gene mutations in the *SRSF2*, *ASXL1*, and *RUNX1* (S/A/R) panel of the training set. (E) Categorization of patients according to the mutation-adjusted risk score (MARS) of advanced systemic mastocytosis versus the WHO classification. Colored bars represent the WHO risk stratification (*x*-axis) in the context of the stratification on the basis of MARS (represented by the rows). (F) To evaluate the ability of the prognostic scores to predict outcome (with 0.5 indicating no discrimination and 1.0 indicating perfect prediction), the C-index is provided for WHO-based stratification, S/A/R mutation–based stratification, clinical risk score (CRS), and MARS. (*) The mast cell leukemia (MCL) cohort included patients with MCL with or without an associated hematologic neoplasm (AHN). ASM, aggressive systemic mastocytosis; SM, systemic mastocytosis.

In the training set, the *KIT* mutation status was as follows: *KIT* D816V (n = 214, 93%), other *KIT* mutations (n = 6, 2%), and *KIT* mutation negative (n = 11, 5%). The status of additional mutations was assessed in 190 (82%) of 231 patients. At least one additional mutation was observed in 82% of all patients. The most frequently affected genes (in ≥ 5% of patients) were *TET2* (n = 79, 42%), *SRSF2* (n = 75, 39%), *ASXL1* (n = 42, 22%), *RUNX1* (n = 34, 18%), *JAK2* (n = 23, 12%), *NRAS*/*KRAS* (n = 17, 9%), *CBL* (n = 17, 9%), *IDH1*/*2* (n = 9, 5%), *SF3B1* (n = 9, 5%), and *EZH2* (n = 9, 5%). The presence of at least one S/A/R mutation and of two or more S/A/R mutations was documented in 105 patients (55%) and 43 patients (23%), respectively (Table 2 and Figs 1A to 1D). An aberrant karyotype was detected in 27 (16%) of 168 patients. With the exception of different numbers of patients without *KIT* mutations (5% in the training set *v* 12% in the validation set), no significant differences were observed between the training and validation sets (eg, the number of S/A/R-positive patients was comparable; Table 2).

**TABLE 2.**
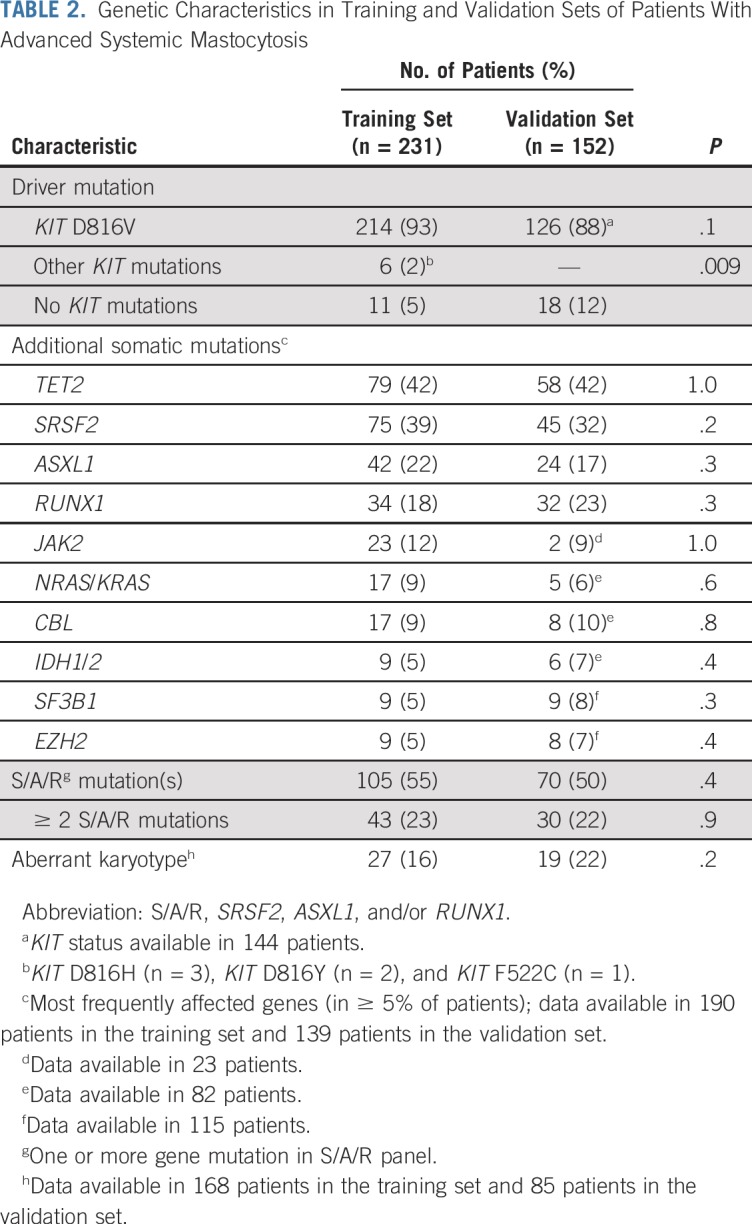
Genetic Characteristics in Training and Validation Sets of Patients With Advanced Systemic Mastocytosis

### Prognostic Impact of the WHO Classification

**FIG A2.**
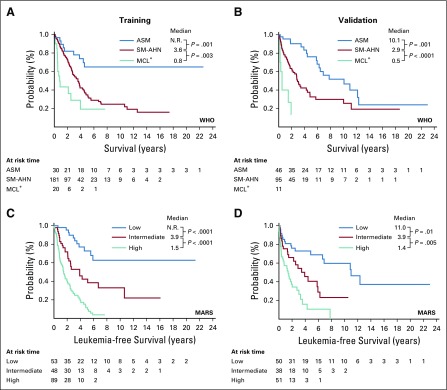
Overall survival and leukemia-free survival for the training set (left) and the validation set (right) of patients with advanced systemic mastocytosis (SM). Patients in both sets are grouped by (A-B) World Health Organization (WHO)–based stratification or (C-D) mutation-adjusted risk score (MARS). (*) The mast-cell leukemia (MCL) cohort included patients with MCL and MCL-AHN. ASM, aggressive SM; MCL-AHN, mast-cell leukemia with an associated hematologic neoplasm; SM-AHN, SM with an associated hematologic neoplasm.

**FIG A3.**
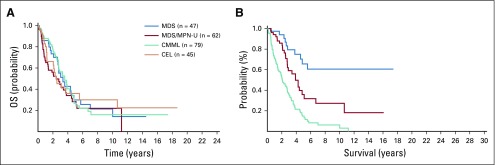
Overall survival (OS) of 233 patients with advanced systemic mastocytosis (SM) with an associated hematologic neoplasm (SM-AHN). (A) grouped by the most common AHN subtypes: myelodysplastic syndrom, MDS, n = 47; MDS/myeloproliferative neoplasm unclassifiable (MDS/MPNU), n = 62; chronic myelomonocytic leukemia (CMML), n = 79; chronic eosinophilic leukemia (CEL), n = 45. (B) OS of patients with SM-AHN was stratified by the mutation-adjusted risk score (MARS).

The WHO classification of AdvSM is of prognostic significance. In the training and validation sets, the median OS times were not reached and 10.1 years for ASM, 3.6 and 2.9 years for SM-AHN, and 0.8 and 0.5 years for MCL (with or without AHN), respectively. The WHO-defined intermediate-risk category of SM-AHN (n = 275, 72%) represented by far the largest group, compared with the low-risk category of ASM (n = 77, 20%) and the high-risk category of MCL (n = 31, 8%; Appendix Figs A1B and A2A-B).

### Prognostic Impact of the S/A/R Gene Panel

**FIG 2.**
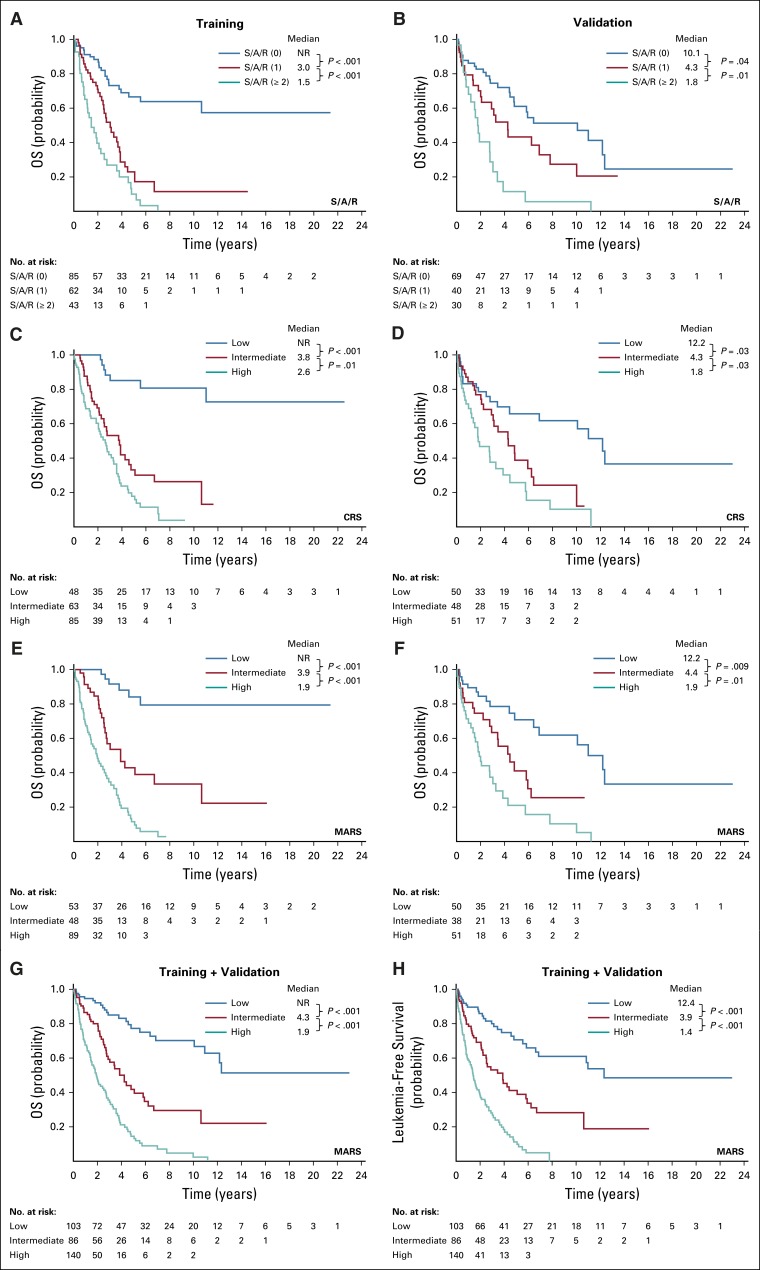
Overall survival (OS) for the training set (left) and the validation set (right) of patients with advanced systemic mastocytosis (AdvSM). Patients in both sets are grouped by (A and B) *SRSF2*, *ASXL1*, and *RUNX1* (S/A/R) mutation–based stratification, (C and D) the clinical risk score (CRS), and (E and F) the mutation-adjusted risk score (MARS) (G) OS and (H) leukemia-free survival of all patients with AdvSM (training and validation sets) by MARS. NR, not reached.

Stratification on the basis of the presence and number of high molecular risk gene mutations (ie, S/A/R) was of significant prognostic impact. In the training and validation sets, median OS times were not reached and 10.1 years for no mutations in the S/A/R panel, 3.0 and 4.3 years for one mutation in the panel, and 1.5 and 1.8 years for two or more gene mutations in the panel, respectively. The three S/A/R-based risk groups were balanced as follows: low risk, 154 patients (47%); intermediate risk, 102 patients (31%); and high risk, 73 patients (22%; Figs 2A and 2B; Appendix Fig A1C).

### Development and Validation of CRS for AdvSM

We applied a Cox proportional hazards model using the patients from the German Registry on Disorders of Eosinophils and Mast Cells in the training set (n = 231). In univariable analyses, the model included the following variables: age greater than 60 years, sex, WHO subtype, hemoglobin less than 10 g/dL, platelets less than 100 × 10^9^/L, mast cell infiltration in BM histology greater than 30%, serum tryptase greater than 150 µg/L, albumin less than 35 g/dL, alkaline phosphatase greater than the upper normal limit (UNL), and splenomegaly (palpable or radiographic, yes or no). The multivariable analysis identified the following four independent predictors of survival: age greater than 60 years (HR, 3.2; 95% CI, 1.8 to 5.9; *P* < .001), hemoglobin less than 10 g/dL (HR, 2.0; 95% CI, 1.3 to 3.0; *P* = .002), platelets less than 100 × 10^9^/L (HR, 1.7; 95% CI, 1.1 to 2.6; *P* = .01), and alkaline phosphatase greater than UNL (HR, 1.8; 95% CI, 1.1 to 2.9; *P* = .03). For assignment of individual scores, we divided the HR value of each variable by the median value of the regression coefficients of all variables in the final model (rounded to nearest 0.5 point). Accordingly, a weighted score of 1 was assigned to hemoglobin less than 10 g/dL, platelets less than 100 × 10^9^/L, and alkaline phosphatase greater than UNL, whereas a score of 1.5 was assigned to age greater than 60 years. On this basis, we generated the CRS, follows: low risk, 0 to 1.5 points; intermediate risk, 2 to 2.5 points; and high risk, 3 to 4.5 points. The model was then applied to the validation cohort (Table 3). The median OS times for the training and validation sets were not reached and 12.2 years for the low-risk group (n = 98, 28%), 3.8 and 4.3 years for the intermediate-risk group (n = 111, 32%), and 2.6 and 1.8 years for the high-risk group (n = 136, 39%), respectively (Table 3, Figs 2C and 2D, and Appendix Fig A1D).

**TABLE 3.**
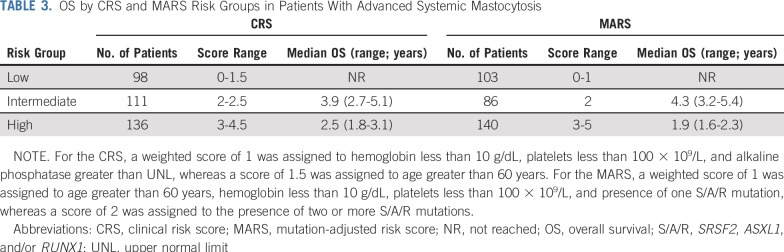
OS by CRS and MARS Risk Groups in Patients With Advanced Systemic Mastocytosis

### Development and Validation of MARS

To appreciate the value of adding molecular information to the CRS, we applied a Cox proportional hazards model to patients for whom mutation status (including S/A/R gene status) was available (training set, n = 191). The model was started by considering the same variables used in developing the CRS and included the presence and number of high molecular risk gene mutations (zero, one, or two or more S/A/R mutations).

Table 4 lists the results of univariable and multivariable analyses in the training set. The multivariable model identified the following five independent predictors of survival: age greater than 60 years (HR, 2.4; 95% CI, 1.4 to 5.0; *P* = .003), hemoglobin less than 10 g/dL (HR, 2.0; 95% CI, 1.3 to 3.0; *P* = .002), platelets less than 100 × 10^9^/L (HR, 1.7; 95% CI, 1.1 to 2.5; *P* = .02), presence of one S/A/R mutation (HR, 2.5; 95% CI, 1.6 to 4.5; *P* < .001), and presence of two or more S/A/R mutations (HR, 4.4; 95% CI, 2.1 to 7.3, *P* < .001). For assignment of individual scores, we divided the HR value of each variable by the median value of the regression coefficients of all variables in the final model (rounded to the nearest 0.5 point). Accordingly, a weighted score of 1 was assigned to age greater than 60 years, hemoglobin less than 10 g/dL, platelets less than 100 × 10^9^/L, and presence of one S/A/R mutation, whereas a score of 2 was assigned to the presence of two or more S/A/R mutations. These weighted scores were used to generate the following three risk groups, which compose the MARS: low-risk group, 0 to 1 point; intermediate-risk group, 2 points; and high-risk group, 3 to 5 points. The model was then applied to the validation cohort. Table 3 lists the OS times of the combined training and validation sets for the CRS and MARS.

**TABLE 4.**
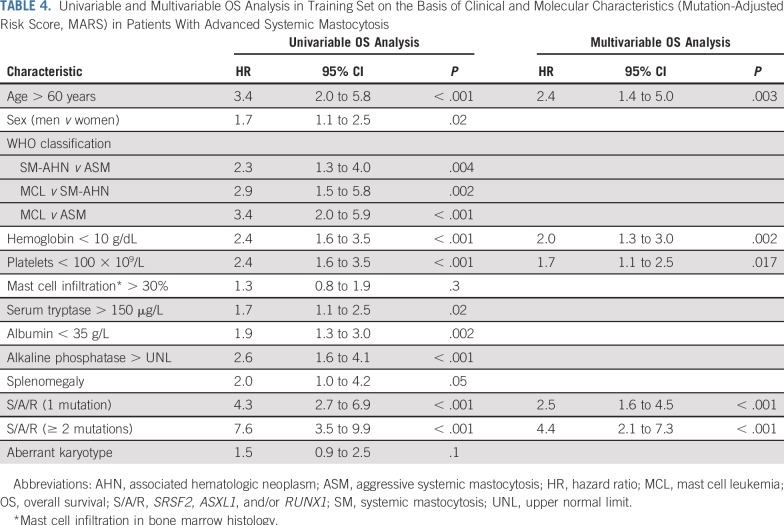
Univariable and Multivariable OS Analysis in Training Set on the Basis of Clinical and Molecular Characteristics (Mutation-Adjusted Risk Score, MARS) in Patients With Advanced Systemic Mastocytosis

The median OS times for the training and validation sets were not reached and 12.2 years for the low-risk group (n = 103, 31%), 3.9 and 4.4 years for the intermediate-risk group (n = 86, 26%), and 1.9 and 1.9 years for the high-risk group (n = 140, 43%), respectively (Table 3 and Figs 2E to 2G). The MARS was also predictive for LFS. The median LFS times for the training and validation sets were not reached and 11 years for the low-risk group, 3.9 and 3.9 years for the intermediate-risk group, and 1.5 and 1.4 years for the high-risk group, respectively (Fig 2H, Appendix Fig A2C and A2D, and Appendix Table A2, online only).

### Comparison of WHO Classification, CRS, and MARS

On the basis of ROC curve analyses, the C-index was 0.42 for the WHO classification, 0.73 for the CRS, and 0.81 for the MARS (Fig 1F). For better comparison of the C-index between the four stratification tools (WHO, S/A/R, CRS, and MARS), we included the same samples (with fully available dataset from the training set, n = 190) across all rules. We established a cross table illustrating the distribution of patients with AdvSM in the new scoring system compared with the WHO classification (Fig 1E). Figure 1E illustrates significant risk redistributions when using MARS across the WHO classification. In particular, the large SM-AHN cohort (n = 237, 72% of all patients) defined as intermediate risk according to the WHO classification was reclassified as low risk (n = 60, 25%), intermediate risk (n = 64, 27%), and high risk (n = 113, 48%) by the MARS. In ASM and MCL (with or without AHN), 38% (n = 24) and 83% (n = 25) of patients were represented in the intermediate-risk and high-risk MARS categories, respectively. The significant advantages of MARS compared with CRS were the enhanced stratification regarding OS within all three risk groups, especially of the intermediate-risk and high-risk groups (Figs 2C to 2F and Appendix Fig A1D), and the prediction of LFS because S/A/R positivity (included in the MARS) at initial diagnosis is significantly associated with transformation to secondary MCL and AML. Seventy percent of all patients with leukemic transformation (n = 42) and available S/A/R status (n = 60) had at least one S/A/R mutation at initial diagnosis.

## DISCUSSION

In clinical practice, the 2016 WHO classification of SM is widely used for prognostic purposes because of the lack of validated international risk scores. Although it robustly distinguishes indolent SM from AdvSM, its value for stratification within the various subtypes of AdvSM (OS: ASM > SM-AHN > MCL) remains suboptimal for the following three reasons: the clinical and histologic heterogeneity represented by the various subtypes of AdvSM; the imbalance of the various subtypes, with SM-AHN representing 70% to 80% of patients and ASM and MCL representing only 20% to 30% of patients; and the wide range of survival times within the subtypes of AdvSM and, in particular, within the SM-AHN variant between a few months and several years.^7,8,11,16,17^ Therefore, the main goal of the current study was to devise and validate a new WHO-independent risk score for patients with AdvSM that integrates objective clinical and mutation characteristics.

The current analysis corroborates the prognostic value of the previously identified high molecular risk gene mutations,^8,9,13,14,18^ especially the negative impact of S/A/R mutations. The presence and number of gene mutations in the S/A/R panel had a strong adverse impact on OS in both the training set and the validation set. The three genes (S/A/R) are among the top five most frequent mutations observed in AdvSM (and also other myeloid neoplasms)^19-21^ and allow a balanced stratification into three risk cohorts.

Next, we established a CRS by defining the following four easily accessible and objective parameters on the basis of multivariable analyses: age greater than 60 years, anemia (hemoglobin < 10 g/dL), thrombocytopenia (platelets < 100 × 10^9^/L), and elevated alkaline phosphatase (> UNL). As illustrated in Figures 2C and 2D and Appendix Figure A1D, LFS and OS were significantly different among the three risk groups. The prognostic impact of the CRS was confirmed in the validation set. The C-index of the CRS was comparable with that of the S/A/R-based stratification (0.73 *v* 0.74, respectively).

Finally, we combined the clinical and molecular data and generated the MARS. In multivariable analyses, age greater than 60 years, anemia (hemoglobin < 10 g/dL), thrombocytopenia (platelets < 100 × 10^9^/L), the presence of one S/AR mutation, and the presence of two or more S/A/R mutations were independent predictors for OS. On the basis of these five parameters, a simple risk scoring system was established for OS. The MARS was confirmed in the validation set and categorizes patients with AdvSM into three groups of significant size. OS times were not reached, 4.3 years, and 1.9 years for low-risk, intermediate-risk, and high-risk patients with AdvSM, respectively. According to the C-index (0.81), the MARS improves the prediction of OS compared with the WHO classification (C-index, 0.42) and the CRS (C-index, 0.73), especially for the intermediate-risk and high-risk groups. In addition, the MARS uses clinical and molecular data that are now commonly available. S/A/R positivity at initial diagnosis, which is the backbone of the MARS, is significantly associated with secondary leukemic transformation (MCL and AML), and therefore, the MARS is also predictive for LFS.

Some recently published risk scores from our own group and from others also included variables such as anemia, thrombocytopenia, elevated alkaline phosphatase, and high molecular risk gene mutations.^10,22^ The pivotal strengths of the current analyses include the following: indolent SM was excluded in the prognostic models because it has a nearly normal life expectancy; this analysis included the greatest number of clinically, morphologically, and genetically well-characterized patients with AdvSM ever reported; most patients had access to targeted treatment modalities such as midostaurin; and the vast majority of patients in the training set were diagnosed through fully centralized pathology and genetic analyses. An addition strength was the homogenous mutation profile (clinical and outcome characteristics) of the training set and the large and independent validation set (derived from centers with expertise in mastocytosis), particularly regarding the individual frequency of gene mutations in the S/A/R panel.

Although there are no data from clinical trials, the MARS may become useful for guiding selection of and predicting response to therapies. Previous data have shown that the multikinase/KIT inhibitor midostaurin has disease-modifying activity in AdvSM, with sustained responses and more favorable outcome in patients with absence of mutations in the S/A/R gene panel and at least 25% reduction of the *KIT* D816V expressed allele burden after 6 months of therapy.^9,16,23,24^ Because the MARS low-risk cohort reflects the majority of these patients, midostaurin may be an optimal choice for these individuals. The generally poor prognosis of MARS intermediate- and high-risk patients may predict less robust responses with currently available therapies, including midostaurin monotherapy, highlighting the need for disease-modifying treatments in these higher risk cohorts.^9,16,23-25^ Because of the significantly higher rates of leukemic transformation and inferior survival, more intensive treatment (eg, combination therapies with midostaurin that also target the AHN or use of more potent and selective second-generation *KIT* D816V inhibitors, followed by allogeneic stem-cell transplantation in eligible candidates) should be considered in these patients. In the largest yet reported cohort of 57 patients with AdvSM undergoing allogeneic stem-cell transplantation, treatment‐related mortality was generally similar to other hematologic neoplasms. Important details included the superior outcome of myeloablative versus dose-reduced conditioning and the heterogenous survival within AdvSM, which was significantly better in SM-AHN compared with ASM or MCL. However, more data are needed, preferably generated in national and international registries, on the key questions regarding optimal timing, debulking, and conditioning strategies.

We conclude that the WHO classification remains the pivotal diagnostic tool for subtyping of SM into indolent SM and AdvSM. The MARS is a WHO-independent and complementary tool for the heterogeneous cohort of patients with AdvSM that defines three risk groups on the basis of a five-parameter risk score and that may improve up-front treatment stratification for these rare hematologic neoplasms.

## Figures and Tables

**TABLE A1.**
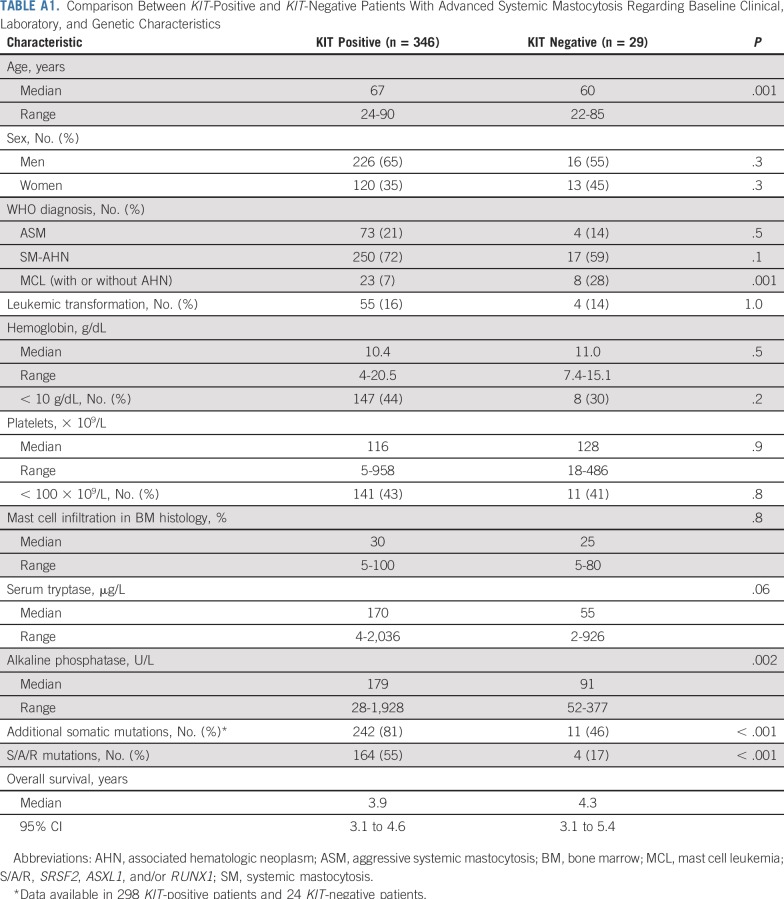
Comparison Between *KIT*-Positive and *KIT*-Negative Patients With Advanced Systemic Mastocytosis Regarding Baseline Clinical, Laboratory, and Genetic Characteristics

**TABLE A2.**
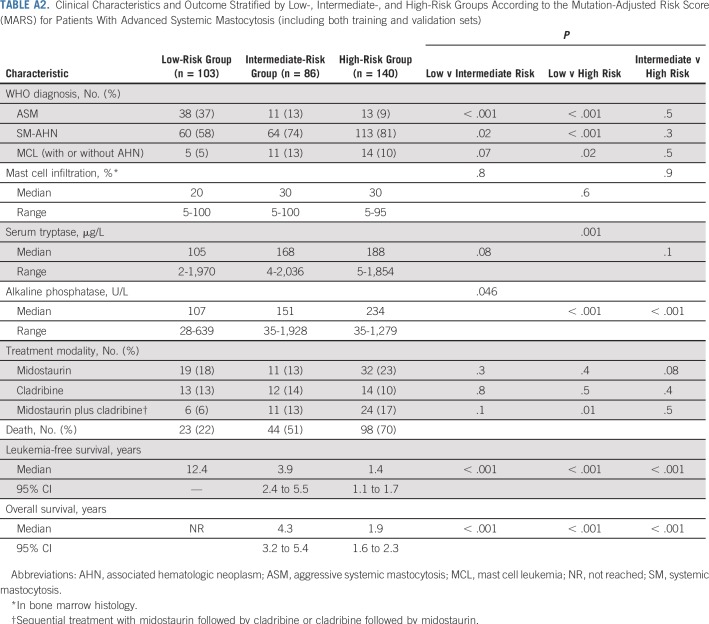
Clinical Characteristics and Outcome Stratified by Low-, Intermediate-, and High-Risk Groups According to the Mutation-Adjusted Risk Score (MARS) for Patients With Advanced Systemic Mastocytosis (including both training and validation sets)
